# Understanding the interaction among enablers of quality enhancement of higher business education in Pakistan

**DOI:** 10.1371/journal.pone.0267919

**Published:** 2022-05-26

**Authors:** Kashif Abbass, Muhammad Asif, Abdul Aziz Khan Niazi, Tehmina Fiaz Qazi, Abdul Basit, Faroq Abdulkarem Al-Muwaffaq Ahmed

**Affiliations:** 1 School of Economics and Management, Nanjing University of Science and Technology, Nanjing, China; 2 Riphah School of Business and Management, Riphah International University, Lahore, Pakistan; 3 Department of Economics & Business Administration, University of Education Lahore, Multan Campus, Multan, Pakistan; 4 Institute of Business & Management, University of Engineering and Technology, Lahore, Pakistan; 5 Hailey College of Banking and Finance, University of the Punjab, Lahore, Pakistan; 6 Lahore Institute of Science & Technology, Lahore, Pakistan; 7 Faculty of Administrative Science, Taiz University, Taiz City, Yemen; University of Defence in Belgrade, SERBIA

## Abstract

This study aims to generate a list of enablers of quality enhancement of higher business education in Pakistan and build a structural model of enablers to prioritize them. It also intends to impose direction and hierarchy on the inter-relationships of the enablers. The study’s design consists of a literature review, data collection from primary sources, and qualitative analysis. Interpretive Structural Modeling (ISM) coupled with Matriced’ Impacts Cruise’s Multiplication Appliquée a UN Classement (MICMAC) is used as a research methodology. The classical procedure of ISM and MICMAC is applied to primary data collected by a field survey from a panel of experts recruited from folks of stakeholders of business education. Results of the literature show that eighteen critical enablers enhance the quality of higher business education in Pakistan. Results of ISM show that the enabler ’job placement of graduates’ occupies the top-level of the ISM model being least critical. In contrast, the enabler ’intra-academia linkages’ occupying the bottom of the model is the most vital. Results of MICMAC show that all enablers, except ’job placement of graduates, are classified into linkage clusters, whereas ‘job placement of graduates’ is classified as an independent cluster. Overall results of the study show that enablers of quality enhancement of higher business education in Pakistan are agile and not settled. The study has profound theoretical, managerial, and practical implications for all stakeholders of business education. It also provides a research framework for future studies concerning subject phenomena. The discussion about the structural model culminates into policy guidelines for the regulators. The study is subject to some methodological/data/resources limitations like the limited review of literature, collection of data from a medium-size panel of experts from Pakistan only, using majority rule for aggregating responses, answering only that what is related to what, other common limitations of qualitative studies, shot period and absence of financial support. The authors conduct this study in a real-life field setting is built on the original dataset and address the efficient issue of phenomenon understudy differently. It is theory-building research, therefore, does not require prior theory. It exploits simple elementary concepts of Boolean algebra, set theory, and graph theory that generates new in-depth information for stakeholders.

## 1. Introduction

Education is considered a hallmark of a nation’s development. In the current regimes, higher education is a significant contributor to the economic development of the countries. The building blocks of higher education have gained fundamental importance over the period. The list of stakeholders of higher education is increasing day by day. There are multiple stakeholders in higher education viz: governmental agencies, faculty members, current students, potential students, parents, competitors, suppliers, administration, accreditation agencies, media, enterprises, the general public, alumnus, and local community [1]. These stakeholders have many concerns with the quality of higher education, and these concerns have been documented in the literature by many studies, e.g. [2, 3]. The students (current and potential), parents, higher education regulators have direct and material concerns about the quality of higher education. Quality of education, in general, and quality of higher education, in particular, is the hot and current schedule of research of the domain. Higher business education has become a fundamental building block of higher education. It is ever-important over the period, and it is now imperative to investigate the issues of quality business education, particularly in developing countries [4]. Examined quality, problems, and indicators of the quality in higher education of Pakistan compared to Germany and concluded that there is a severe need to enhance the quality of education in Pakistan [5]. Asserted that Pakistan is struggling to improve the quality of higher business education even though its business schools are claiming the high professional competencies of its business graduates. Undoubtedly, there is an influx of literature on this behalf but hardly can find any study on the enablers of quality of higher business education. The term quality education is relatively confusing in literature, and it is hard to find consensus about it [2, 6]. However, it is evident that to embark on quality higher education; the stakeholders have to eliminate the systemic weaknesses and exploit the systemic strengths [7]. The stakeholders of business education are also striving to bridge up the academia-industry linkage. It is considered high time now to ascertain the enablers of quality business education [5, 8]. Therefore, the study focuses on this research gap, particularly that of structure underlying the enablers of enhancing the quality. Hence, the scope of the study is to explore enablers of quality enhancement of higher business education in Pakistan. The study is helpful for governments, regulators, management of academic institutions/competitors, accreditation agencies, faculty, current/potential/alumnus students and parents, local and research community. Therefore, the results of the study are generalizable for stakeholders of business education in Pakistan. Objectives of the study are: i) to identify, hierarchical, and classify the enablers, ii) to build an interpretive model, and iii) to discuss the model and its implications in reality. To achieve the objectives, a wide array of methodological choices [9] are considered, and Interpretive Structural Modeling (ISM) is found to be appropriate. ISM is a handy tool for qualitative analysis. It is suitable for analyzing complex interdependent relationships in entangled and rapidly changing situations. It can articulate the complexities into visible, well-defined models with graphical presentations [10–12].

**Fig 1:**
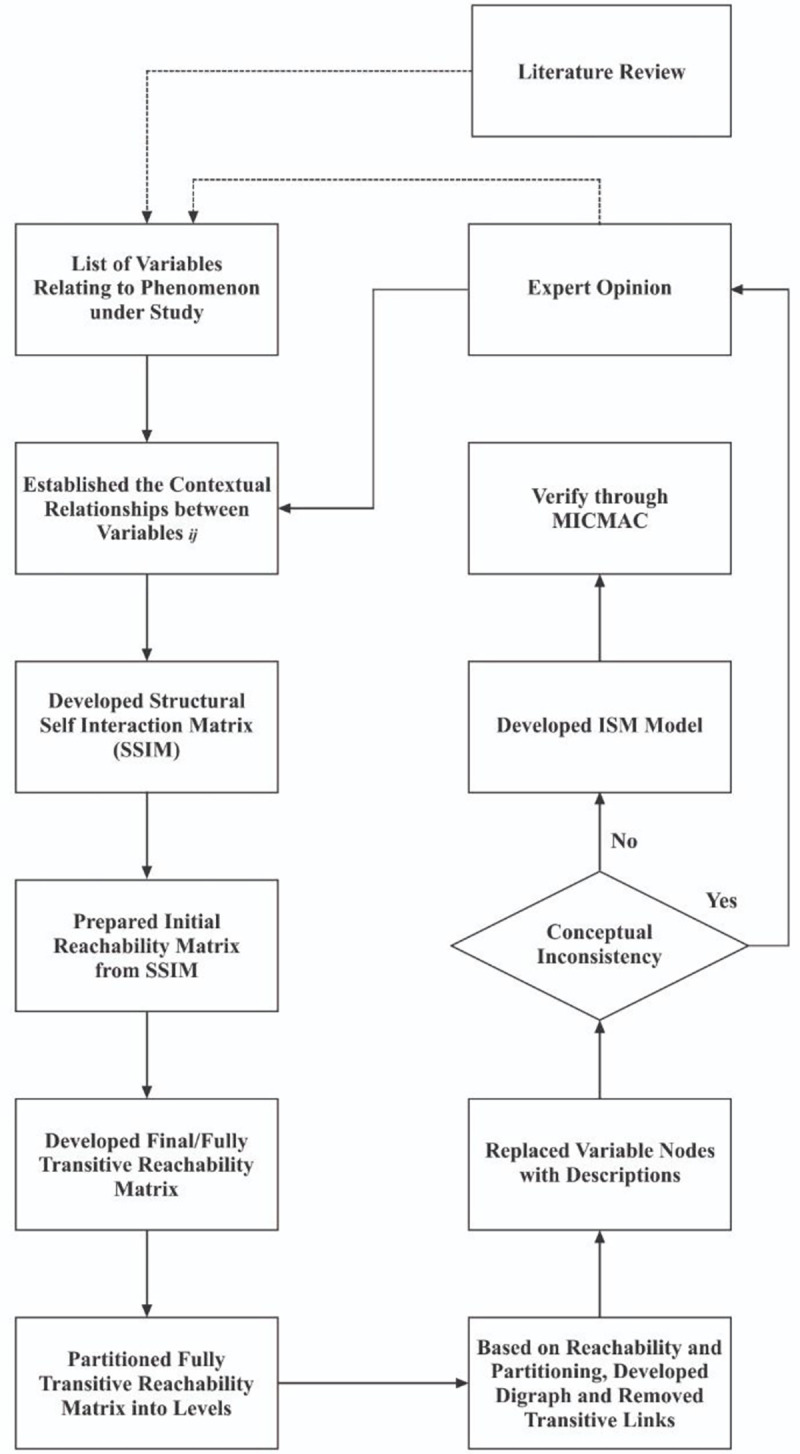
Schema of ISM methodology. Source: [13].

Since the phenomenon under study is a complex phenomenon, the ISM method is justified and the most appropriate one. It outperforms statistical approaches in the cases like that presented in our hand. ISM, in general, and, in this case, has an advantage over other methodologies because it is a rigorous mathematical model exchange isomorphism technique. It can transform the mental models into binary models and then convert them into graphical models to simplify the complex inter-factor relations by eliminating the redundant ones [10]. Triangulation of ISM and MICMAC is also expected and useful since MICMAC has the capability of verifying and/or corroborating the results of ISM. As a result, the study adds a structural model and a classification diagram based on the driving dependence of enablers in theory apart from valuable simplified information for practitioners. The classical procedure of ISM is devised by [10] and used by [13] is adopted (Fig 1).

The study is divided into five sections, i.e., the introduction being section one, literature review section two, methodology/data collection & analysis section three, results & discussion section four, and conclusion section five.

## 2. Literature review

Before going through any data collection and analysis, the authors have surveyed the relevant contemporary literature available online in world-renowned databases (say, Emerald, ScienceDirect, Wiley-Blackwell, JStor, Taylor & Francis, Ebscohost, etc.) using Google as a search engine. There is a lot of literature on quality education, e.g., quality issues of higher educational institutions of Slovak Republic [14], quality assurance, and regulating of higher education in Hong Kong [15], development of higher education quality assessment model at King Abdul-Aziz University, KSA [16], implementation of education on dual-use in four countries: Pakistan, Italy, Sweden, and Austria [17], service quality playing a mediating role between quality management enablers and students’ satisfaction in higher education institutions in Iran [18], quality assurance in higher education institutions of China [19]. Likewise, there is a plethora of research on the issues, directly and indirectly, concerned with the quality of higher education. e.g. [20] carried out a comprehensive study to conceptualize the challenges faced by European higher education in the implementation of quality in the education sector and classified them into three categories i) quality culture and leadership challenges, ii) implementation challenges (execution, funding, and competency) and iii) organization challenges (education system, quality system, and external stakeholders). Uncut literature may not be possible to accommodate in the study; however, relevant studies are cited here [21]. Highlighted the need for innovative quality enhancement frameworks and institutional collaborative networks for quality assurance and equality enhancement in engineering education [22]. Concluded a Quality Culture Inventory (QCI) consists of leadership, quality-focused commitment, and communication that permits higher education institutions to assess their quality culture. A lot of research surpassed on higher education sector across the globe, including analysis of the current system of quality assurance and recommendations of quality enhancement of business management education in Africa [23], challenges being faced by European higher education in the implementation of quality in education sector [20], analysis of the quality audit report of Australian universities to ascertain quality assurance issues [24], teacher education and their widening participation: a case of England and Australia [25], productivity an indicator of the quality of Greece higher education system [26], quality assurance and student experience in higher education: the case of West Indies [27]. [28] Studied three university courses as six sigma, quality management, and lean production. They analyzed each class to determine the factors affecting students’ perceived quality and learning of the respective courses. They argued that course design considerably affects students’ course evaluation and learning [29]. Asserted that continued commitment is required to plummet gender inequalities in UK higher education. [17] researched four countries: Pakistan, Sweden, Italy, and Austria. They stated that no such approach as "one size fits all" prevails in the enactment of quality of education [30]. Argued that better augmentation of digital technologies in higher education to achieve academic success and support students’ understanding of four inter-related factors (i.e., organization, information, social arrangements, and technology) is essential to enhance the quality of education.

### 2.1 Scenario of higher business education in Pakistan

Apart from science and technology, business education also attained a high degree of attention in Pakistan. For the last two decades, the Pakistan government has put tremendous effort into embarking on the regime of high-quality education. A national-level educational framework meeting international standards have been introduced through the higher education commission. It is of serious concern for the international community, the Government of Pakistan, and Pakistani society at large to somehow evaluate & enhance the quality of business education in Pakistan. There are a lot of research studies in this context as well, e.g. [31] affirmed that there are four chief factors such as leadership barrier, culture barrier, internal barrier, and external barrier to adopting new specialized courses [32]. Evaluated the relationship between web-based services and distance education students’ satisfaction. They revealed no significant difference among males and females regarding their satisfaction with the use of web-based services in distance education [33]. Analyzed the academic dishonesty of higher education institutions of Pakistan and documented the impact of that situational, individual, and ethical factor on academic dishonesty [34]. Conducted a qualitative study to understand the women’s viewpoint and experiences regarding their careers and progress working in the University of the Northern City of Khyber Pakhtunkhwa, province of Pakistan, and proclaimed that it is quite challenging to maintain a balance between work and home responsibilities [35]. Held a comprehensive study to assess the education quality of private sector universities compared to Pakistan’s public sector universities [36]. Asserted that the knowledge management process significantly impacts directly and indirectly on organizational performance through intellectual capital and innovation in higher education institutions [37]. Bolstered that dearth of suitable teaching materials, low registration at the primary level, inadequate physical infrastructure, lack of skilled/trained faculty, and vast disparity between gender and regions show the poor performance of the education sector [38]. Proposed a model that provides entrepreneurial mindsets for managing higher education institutions in Pakistan to enhance the quality of entrepreneurial education and research [39]. Emphasized the need for digitization in the higher education sector of Pakistan by way of using digital media for the acquirement of information resources [8]. Examined the relationship between students’ expectations and perceptions of perceived education service quality and found a substantial gap. To the best of the authors’ knowledge, one can hardly find a study that addresses the issue of enhancement of quality higher business education in totality; however, from the survey of the literature, the authors identified the eighteen enablers for enhancing quality in business education from dispersed literature (Table 1).

**Table 1:** List of enablers for enhancing quality in higher business education.

Code	Enablers	Description	Source
**1**	Appropriate Funds for Research	Funding for scientific business research by government/ regulators, Higher Education Institutions (HEIs), industry, or NGOs/international institutions.	[20]
**2**	Pro-Research Environment	Pro-research environment behavior means consciously minimizing the negative factors and promoting positive aspects.	Panel of Experts
**3**	Financial Assistance for Students	Financial support is available to students for furthering their education.	[40]
**4**	Effective implementation of rules and regulations	Ensuring the implementation of rules and regulations for the quality of higher education devised by regulatory bodies.	[27]
**5**	Provision of Infrastructure	Provision of appropriate space, furniture & fixture, connectivity, and access to contemporary research.	[37]
**6**	Learned and Competent Faculty	Qualified, experienced, competent, and dedicated faculty.	[41]
**7**	Availability of State-of-the-Art Technology	Making available the latest software/hardware and high-speed connectivity for researchers (both students and faculty).	[42]
**8**	Industrial Linkages	Students and faculty exposure to industry and arrangements of HEIs for close contact with the industry and commercialization of academic research.	[26]
**9**	Knowledge Sharing Culture	Inculcating the culture of sharing tacit/explicit knowledge among the stakeholders.	[37]
**10**	Topical Curriculum	The topical approach means selecting study topics suitable to an audience’s age, ability, and interest, dealing with the issue entirely after it is introduced first.	[43]
**11**	Intra-Academia Linkages	Intra-academia linkages mean relationships among HEIs at the level of administration, faculty, and students.	[25, 44]
**12**	Job Placement of Graduates	The job placement facility is available to graduating students through academia/industry agreements.	[43]
**13**	Faculty Development & Training	Continuous effort to enhance the skills of faculty by way of training and providing them an opportunity to learn new skills under the faculty development program.	[43]
**14**	Access to Necessary Business Tools	Access to the latest software, research databases, and state-of-the-art digital resources of data and/or digital libraries.	[37]
**15**	Students/Faculty Exchange Program	Faculty and students exchange through teaching, training, conferences, workshops, etc.	[25]
**16**	Research Publication Opportunities	Opportunities created by HEIs, regulators, accreditation bodies for publication of research in journals, conference proceedings, or working papers/reports.	[40]
**17**	Teacher Student Collaboration	Teacher-student collaboration means tandem to lead, instruct, and mentor students that can implement across all instructional levels and subject areas.	[45]
**18**	Business Bodies Accreditation	Business accreditation means achieving internationally recognized standards to demonstrate competence, impartiality, and performance capability.	23, 41]

The enablers identified from literature sources (Table 1) were verified from a panel of experts using the approval vote method with the rule ’minority gives way to the majority.

## 3. Methodology/Data collection and analysis

The study follows post-positivism research philosophy with induction as an approach. It is a qualitative study designed on primary data. The sample size has been decided according to the norms of constituting homogenous/heterogeneous panels of experts for qualitative studies. In this study, the panel consists of sixteen homogenous experts of higher business education. The data has been collected from the experts by using a matrix type VAXO base questionnaire (i.e. (n(n−1))/2 matrix type) [46]. Common methods used to elicit data from experts are Delphi, brainstorming, in-depth discussion, nominal group technique, repertory-grid interview technique, matrix type questionnaire, laddering interview, problem-solving group session, one-to-one, face-to-face in-depth interview, triadic sorting task approach, approval voting on alternatives/elect alternatives, idea engineering workshop and idea generation exercise, etc. This study uses one-to-one, face-to-face, in-depth interviews in combination with approval voting on alternatives for every pair of relations using the VAXO questionnaire as an instrument. The study uses i) review of literature for identification of enablers, ii) classical Interpretive Structural Modeling (ISM) for hierarchicalizing, simplifying & structuring relations, and iii) Matrice d’Impacts Croisés Multiplication Appliquée á un Classement (MICMAC) for analysis and classification. The study is built on basic concepts of Boolean algebra, set theory, and directed graph theory. There are numerous methods of identification of factors viz: Literature Review [47]; Expert Opinion [48–50]; Case Study [12]; Delphi Method [51] Exploratory Factor Analysis [52]; Meta-Analysis [53]; Presumed by Authors [53]; Idea Engineering Workshop and Brainstorming Session [54]; Interview Content analysis [55]; and Anecdotal Evidence from Literature [56]. The study uses the literature review method in combination with experts’ opinions.

### 3.1 Panel of experts

A panel of experts is usually constituted where the data are either not existing, limited, expensive, or unreliable. The data on the phenomenon under investigation particularly about: How experts think to enhance the quality of higher business education? Is not exist. It is appropriate to constitute the panel because it outperforms the statistical groups and provides more valid data than that of statistical collected from masses. According to ISM-based studies’ norms, the panel consists of fifteen to twenty-five experts to gain optimum results [55]. We considered theoretical/expert knowledge and practical experience (i.e., minimum of ten years in authoritative organizations). It is a qualitative study based on an inductive approach having depth instead of breadth. The size of a panel of experts (sample) in this type of qualitative study may be as less as 5–7 experts [12]. Generally, the ideal size for a heterogeneous panel is 8–12 experts and 15–25 experts for homogenous panels. This type of study’s scope is 10–20 experts [46, 57–59]. In this study, the panel size is sixteen experts, i.e., considered to be the optimum size of the panel. The panel consists of one director of quality enhancement cell, one director-level focal person designated by the Higher Education Commission of Pakistan to monitor the quality of education and two professors (holding Ph.D. degrees in business administration) of sizeable public sector universities, two Ph.D. students having 12 years of teaching/working experience, four senior managers holding master degree in business administration and having a hands-on job for 15 years in the industry, and six students of masters in science in business administration having more than ten years of experience currently doing theses in sizeable public sector universities. Rapport was first developed with prospective respondents for briefing about the study. Then they were approached by the researchers in their field setting to get approval vote regarding enablers to be included in the research and finalize the questionnaire matrix. After finishing the questionnaire, approached the expert for data elicitation using the ‘in-depth face-to-face one-on-one interview”. It took us more than two months to collect the data. The data on paired relations of enablers were taken on (*n*(*n*−1))/2 matrix (*ij* part of the questionnaire) separately from each expert. *VAXO* symbols are used to extract data. Questionnaire (S1 Annex) contained instructions for completing the questionnaires, i.e., 1) fill white cells only, 2) contextual relationship = leads to, 3) enter *V* when the row influences the column, 4) enter *A* when the column influences the row, 5) enter *O* when there is no relation between the row and the column and 6) enter *X* when row and column influence each other. The responses were aggregated using the mode, approached expert three times during the conduct of the study. Firstly, for developing the rapport/briefing about the research and approval of enablers; secondly, for data collection; and thirdly, for review of the extracted ISM model too logically, conceptually, and theoretically verification of the model.

### 3.2 Interpretive structural modeling

Classical procedure of ISM, as depicted in Fig 1, is followed. SSIM (Table 2) was developed due to the aggregation of data collected from experts.

**Table 2:** Structural Self-Interaction Matrix (SSIM).

Code.	1	2	3	4	5	6	7	8	9	10	11	12	13	14	15	16	17	18
**1**		V	V	O	V	A	V	O	O	O	A	O	V	V	X	A	X	O
**2**			O	A	A	A	O	A	X	O	O	V	V	V	X	V	X	X
**3**				A	O	A	A	A	O	O	O	V	O	V	A	V	O	V
**4**					O	X	A	O	X	V	X	V	A	V	V	V	X	O
**5**						A	V	V	O	O	V	V	V	O	V	O	V	A
**6**							O	V	A	O	V	O	A	V	V	V	X	O
**7**								V	V	O	V	O	A	A	V	O	O	V
**8**									O	O	O	V	X	A	O	V	O	V
**9**										O	X	O	A	A	A	O	V	O
**10**											X	V	A	O	O	O	A	O
**11**												V	V	A	V	V	X	O
**12**													O	A	A	O	O	A
**13**														V	O	X	V	V
**14**															O	V	V	V
**15**																V	X	O
**16**																	A	X
**17**																		O
**18**																		

SSIM was converted into an initial reachability matrix (Table 3) using the classical procedure of ISM devised by [10] and used by [60].

**Table 3:** Initial reachability matrix.

Code	1	2	3	4	5	6	7	8	9	10	11	12	13	14	15	16	17	18
**1**	1	1	1	0	1	0	1	0	0	0	0	0	1	1	1	0	1	0
**2**	0	1	0	0	0	0	0	0	1	0	0	1	1	1	1	1	1	1
**3**	0	0	1	0	0	0	0	0	0	0	0	1	0	1	0	1	0	1
**4**	0	1	1	1	0	1	0	0	1	1	1	1	0	1	1	1	1	0
**5**	0	1	0	0	1	0	1	1	0	0	1	1	1	0	1	0	1	0
**6**	1	1	1	1	1	1	0	1	0	0	1	0	0	1	1	1	1	0
**7**	0	0	1	1	0	0	1	1	1	0	1	0	0	0	1	0	0	1
**8**	0	1	1	0	0	0	0	1	0	0	0	1	1	0	0	1	0	1
**9**	0	1	0	1	0	1	0	0	1	0	1	0	0	0	0	0	1	0
**10**	0	0	0	0	0	0	0	0	0	1	1	1	0	0	0	0	0	0
**11**	1	0	0	1	0	0	0	0	1	1	1	1	1	0	1	1	1	0
**12**	0	0	0	0	0	0	0	0	0	0	0	1	0	0	0	0	0	0
**13**	0	0	0	1	0	1	1	1	1	1	0	0	1	1	0	1	0	0
**14**	0	0	0	0	0	0	1	1	1	0	1	1	0	1	0	1	1	1
**15**	1	1	1	0	0	0	0	0	1	0	0	1	0	0	1	1	1	0
**16**	1	0	0	0	0	0	0	0	0	0	0	0	1	0	0	1	0	1
**17**	1	1	0	1	0	1	0	0	0	1	1	0	0	0	1	1	1	0
**18**	0	1	0	0	1	0	0	0	0	0	0	1	0	0	0	1	0	1

The initial reachability matrix was checked for transitive relations on scientific bases using functions of MS Excel and identified incorporated transitive ties into the final reachability matrix (Table 4) distinguished by 1*.

**Table 4:** Final reachability matrix.

Code	1	2	3	4	5	6	7	8	9	10	11	12	13	14	15	16	17	18	Driving
**1**	1	1	1	1*	1	1*	1	1*	1*	1*	1*	1*	1	1	1	1*	1	1*	**18**
**2**	1*	1	1*	1*	1*	1*	1*	1*	1	1*	1*	1	1	1	1	1	1	1	**18**
**3**	1*	1*	1	0	1*	0	1*	1*	1*	0	1*	1	1*	1	0	1	1*	1	**14**
**4**	1*	1	1	1	1*	1	1*	1*	1	1	1	1	1*	1	1	1	1	1*	**18**
**5**	1*	1	1*	1*	1	1*	1	1	1*	1*	1	1	1	1*	1	1*	1	1*	**18**
**6**	1	1	1	1	1	1	0	1	1*	1*	1	1*	1*	1	1	1	1	1*	**17**
**7**	1*	1*	1	1	1*	1*	1	1	1	1*	1	1*	1*	1*	1	1*	1*	1	**18**
**8**	1*	1	1	1*	1*	1*	1*	1	1*	1*	0	1	1	1*	1*	1	1*	1	**17**
**9**	1*	1	1*	1	1*	1	0	1*	1	1*	1	1*	1*	1*	1*	1*	1	1*	**17**
**10**	1*	0	0	1*	0	0	0	0	1*	1	1	1	1*	0	1*	1*	1*	0	**10**
**11**	1	1*	1*	1	1*	1*	1*	1*	1	1	1	1	1	1*	1	1	1	1*	**18**
**12**	0	0	0	0	0	0	0	0	0	0	0	1	0	0	0	0	0	0	**1**
**13**	1*	1*	1*	1	1*	1	1	1	1	1	1*	1*	1	1	1*	1	1*	1*	**18**
**14**	1*	1*	1*	1*	1*	1*	1	1	1	1*	1	1	1*	1	1*	1	1	1	**18**
**15**	1	1	1	1*	1*	1*	1*	0	1	1*	1*	1	1*	1*	1	1	1	1*	**17**
**16**	1	1*	1*	1*	1*	1*	1*	1*	1*	1*	0	1*	1	1*	1*	1	1*	1	**17**
**17**	1	1	1*	1	1*	1	1*	1*	1*	1	1	1*	1*	1*	1	1	1	1*	**18**
**18**	1*	1	0	0	1	0	1*	1*	1*	0	1*	1	1*	1*	1*	1	1*	1	**14**
**Dependence**	**17**	**16**	**15**	**15**	**16**	**14**	**14**	**15**	**17**	**15**	**15**	**18**	**17**	**16**	**16**	**17**	**17**	**16**	**286**

A fully transitive reachability matrix was then partitioned (S1-S6 Tables in S2 Annex) by applying the standard iteration method as devised by [10]. The iterations as represented in S1-S6 Tables in S2 Annex are summarized as S7 Table in S2 Annex. Using the permutation method devised by [10], a conical matrix is prepared as S8 Table in S2 Annex. The grey cells indicate the extraction of the ISM model on diagonals. For brevity, an abridged representation of ISM is given here in Table 5.

**Table 5:** Abridged representation of ISM.

Reachability Sets																						
**Antecedent Sets**	**Level**	**Code**	**12**	**1**	**9**	**10**	**13**	**16**	**17**	**18**	**2**	**3**	**5**	**14**	**4**	**6**	**15**	**7**	**8**	**11**		**Driving Power**
	***Level I***	**12**	1	0	0	0	0	0	0	0	0	0	0	0	0	0	0	0	0	0	**1**	
	***Level II***	**1**	1*	1	1*	1*	1	1*	1	1*	1	1	1	1	1*	1*	1	1	1*	1*	**18**	
		**9**	1*	1*	1	1*	1*	1*	1	1*	1	1*	1*	1*	1	1	1*	0	1*	1	**17**	
		**10**	1	1*	1*	1	1*	1*	1*	0	0	0	0	0	1*	0	1*	0	0	1	**10**	
		**13**	1*	1*	1	1	1	1	1*	1*	1*	1*	1*	1	1	1	1*	1	1	1*	**18**	
		**16**	1*	1	1*	1*	1	1	1*	1	1*	1*	1*	1*	1*	1*	1*	1*	1*	0	**17**	
		**17**	1*	1	1*	1	1*	1	1	1*	1	1*	1*	1*	1	1	1	1*	1*	1	**18**	
		**18**	1	1*	1*	0	1*	1	1*	1	1	0	1	1*	0	0	1*	1*	1*	1*	**14**	
	***Level III***	**2**	1	1*	1	1*	1	1	1	1	1	1*	1*	1	1*	1*	1	1*	1*	1*	**18**	
		**3**	1	1*	1*	0	1*	1	1*	1	1*	1	1*	1	0	0	0	1*	1*	1*	**14**	
		**5**	1	1*	1*	1*	1	1*	1	1*	1	1*	1	1*	1*	1*	1	1	1	1	**18**	
		**14**	1	1*	1	1*	1*	1	1	1	1*	1*	1*	1	1*	1*	1*	1	1	1	**18**	
	***Level IV***	**4**	1	1*	1	1	1*	1	1	1*	1	1	1*	1	1	1	1	1*	1*	1	**18**	
		**6**	1*	1	1*	1*	1*	1	1	1*	1	1	1	1	1	1	1	0	1	1	**17**	
		**15**	1	1	1	1*	1*	1	1	1*	1	1	1*	1*	1*	1*	1	1*	0	1*	**17**	
	***Level V***	**7**	1*	1*	1	1*	1*	1*	1*	1	1*	1	1*	1*	1	1*	1	1	1	1	**18**	
		**8**	1	1*	1*	1*	1	1	1*	1	1	1	1*	1*	1*	1*	1*	1*	1	0	**17**	
	***Level VI***	**11**	1	1	1	1	1	1	1	1*	1*	1*	1*	1*	1	1*	1	1*	1*	1	**18**	
			**18**	**17**	**17**	**15**	**17**	**17**	**17**	**16**	**16**	**15**	**16**	**16**	**15**	**14**	**16**	**14**	**15**	**15**		
**Dependence Power**																						

**Fig 2:**
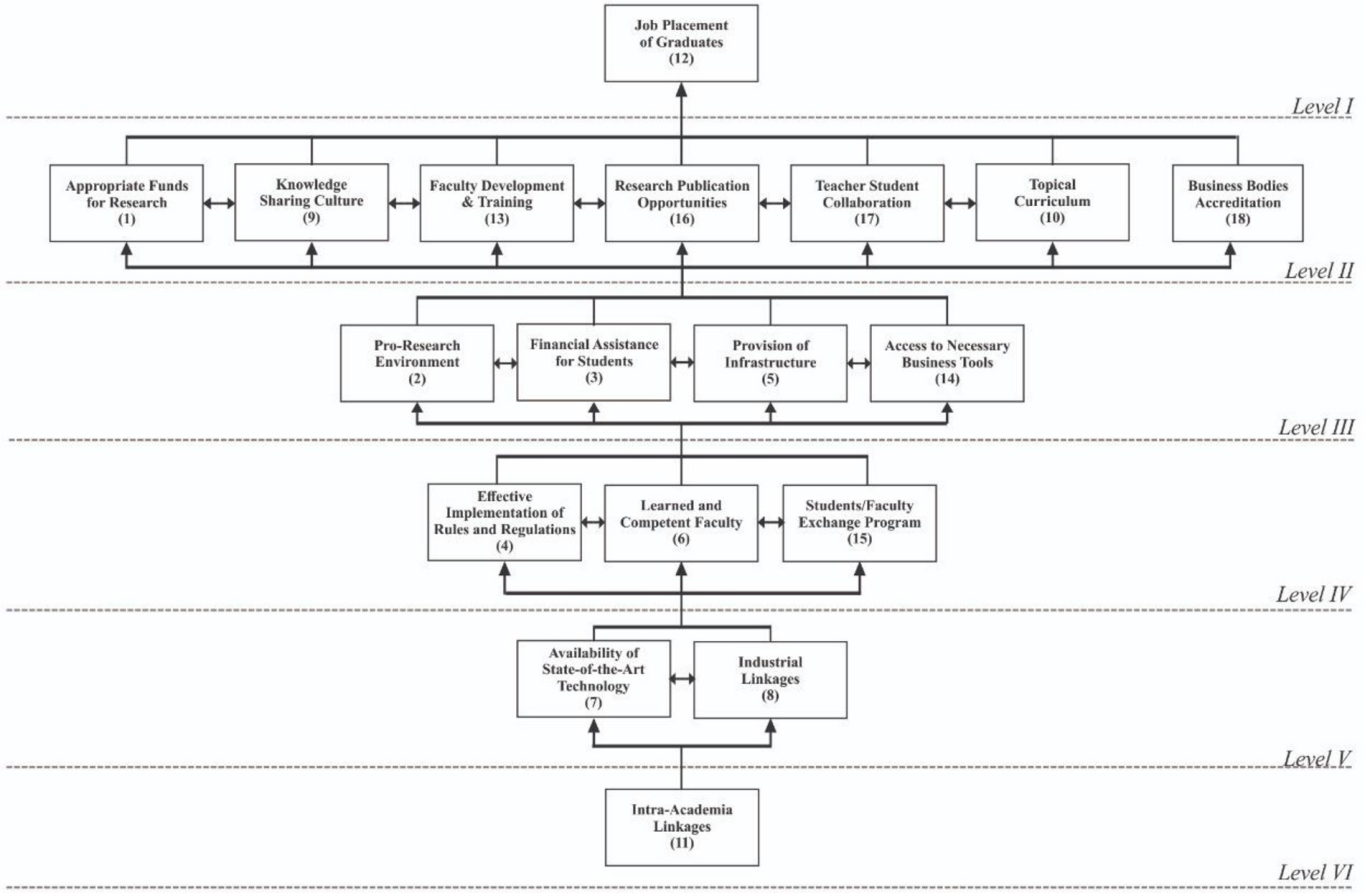
ISM model. Source: Author’s Constructed.

Based on level partitioning as a result of iterations, a directed graph, namely the ISM model (Fig 2), has accordingly been constructed.

Close observation of Fig 2 reveals that enabler 12 occupy the top-level of ISM model (*Level I*); enablers 1, 9, 10, 13, 16, 17, and 18 occupy upper-middle (*Level II*); enablers 2, 3, 5, and 14 (*Level III*) and enablers 4, 6 and 15 (*Level IV*) occupy middle; enablers 7 and 8 (*Level V*) occupy lower-middle level, and enabler 11 occupy bottom (*Level VI*) of ISM model.

### 3.3 MICMAC analysis

**Fig 3:**
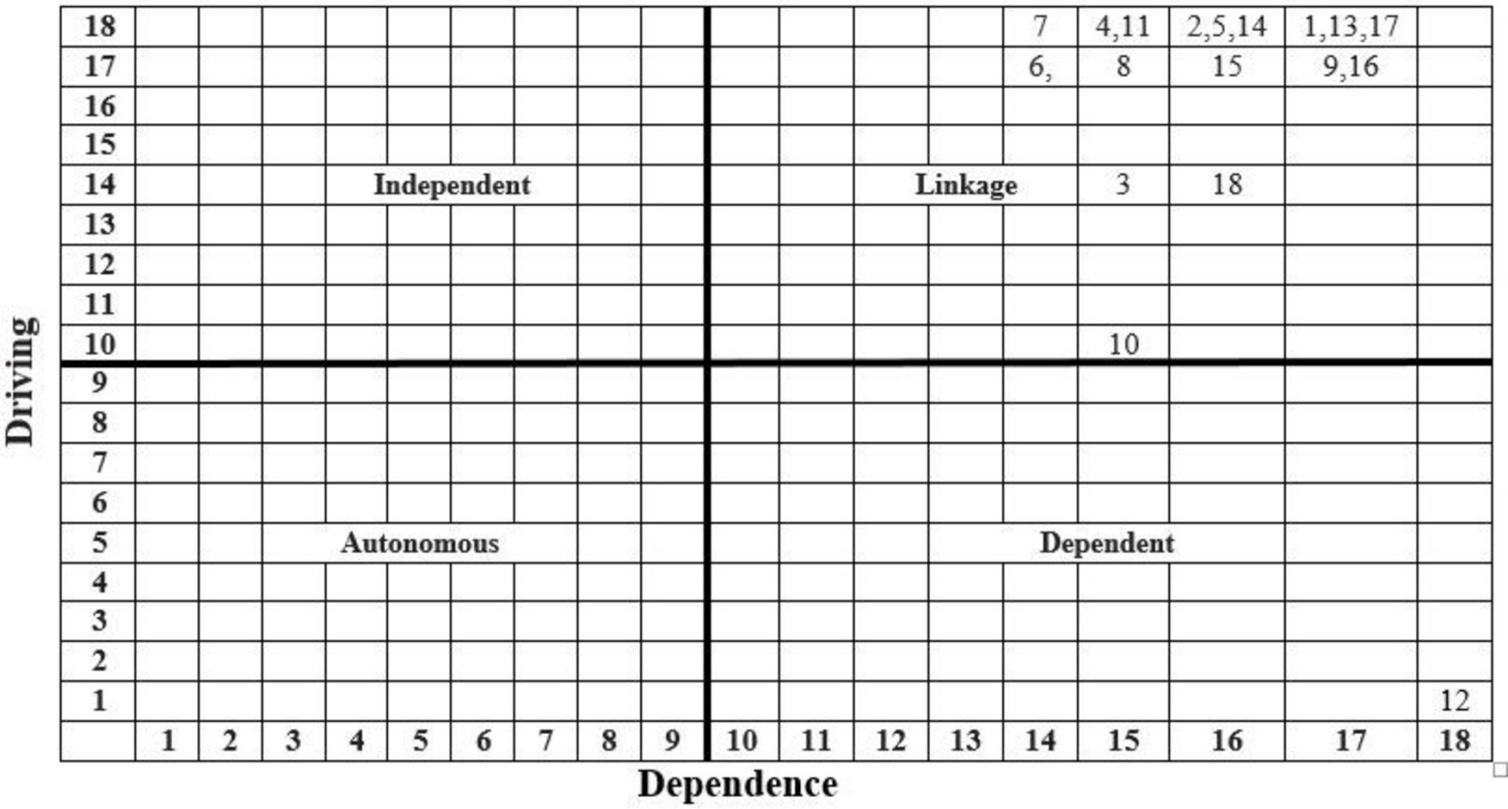
Driving-dependence diagram. Source: Author’s Constructed.

Using a scale-centric approach, a driving-dependence diagram (Fig 3) has been prepared from a transitive reachability matrix (Table 4) by applying conventional procedure MICMAC devised by [61].

The results show that all enablers (1, 2, 3, 4, 5, 6, 7, 8, 9, 10, 11, 13, 14, 15, 16, 17 and 18) except ‘job placement of graduates (12)’ are classified in linkage cluster; that means the elements of the system are agile, ambivalent and unsettled. Enabler ’job placement of graduates’ is classified as dependent, and none of the enablers is classified as autonomous and independent.

## 4. Results & discussion

### 4.1 Results

With the admittance of ‘management’ as a science, higher business education has become a fundamental building bloc. The problem under investigation is identifying, analyzing, and classifying the mystified enablers of quality enhancement of higher business education in Pakistan. In contrast, the study’s objectives are to identify, hierarchical, and organize the enablers thereof, to build an interpretive model using literature review, ISM, and MICMAC methods. Results of the literature show that eighteen vital enablers contribute to the phenomenon of quality of higher business education (Table 1). Results of ISM show that enabler ‘job placement of graduates (12)’ occupy top-level of ISM model (*Level I*); enablers appropriate funds for research (1), knowledge sharing culture (9), topical curriculum (10), faculty development & training (13), research publication opportunities (16), teacher-student collaboration (17) and business bodies accreditation (18) occupy upper-middle (*Level II*); enablers pro-research environment (2), financial assistance for students (3), provision of infrastructure (5) and access to necessary business tools (14) (*Level III*) and enablers effective implementation of rules and regulations (4), learned and competent faculty (6) and students/faculty exchange program (15) occupy middle (*Level IV*); enablers availability of state-of-the-art technology (7) and industrial linkages (8) occupy lower middle (*Level V*), and enabler intra-academia linkages (11) occupy the bottom of the model (*Level VI*). Results of MICMAC classify enablers into four clusters, i.e., *Autonomous*: There is no enabler classified in an autonomous group. In fact, in this cluster, those enablers are classified that having weak driving, weak dependence, separated from the model, having few but strong links, and do not have much impact on the system. This result confirms that all the enablers are essential and relevant to the phenomenon at hand. *Dependent*: Job placement of graduates (12) is classified into the dependent cluster. The enablers classified in this cluster have weak driving but strong dependence on others. It is also believed to be a logically valid result. *Linkage*: The enablers: appropriate funds for research (1), pro-research environment (2), financial assistance for students (3), effective implementation of rules and regulations (4), provision of infrastructure (5), learned and competent faculty (6), availability of state-of-the-art technology (7), industrial linkages (8), knowledge sharing culture (9), topical curriculum (10), intra-academia linkages (11), faculty development & training (13), access to necessary business tools (14), students/faculty exchange program (15), research publication opportunities (16), teacher-student collaboration (17) and business bodies accreditation (18) are classified into linkage cluster. The enablers classified in this cluster have intense driving & dependence. They are unstable, unbalanced, and/or agile; therefore, they can affect others, having a feedback effect on themselves. Where the more enablers fall in this cluster, it can construe that the system is in its infancy and is struggling to make some sense. Therefore, the regulators should focus on linkage factors. This interpretation is accurate for the phenomenon at hand. *Independent*: There is no enabler as such classified into separate clusters, but there are 1, 9, 13, 16, and 17 are classified into linkage clusters they have high driving power and have the potential to be independent, but since they also have strong dependence power, therefore, they are classified into the linkage. The results aforementioned are abridged in eye span as Table 6.

**Table 6:** Juxtaposed results of literature, MICMAC, and ISM.

Result of Literature Review		Results of MICMAC Analysis				Results of ISM	Comments
Code	Issue	Driving	Dependence	Effectiveness	Cluster	Level	
**1**	Appropriate Funds for Research	18	17	1	*Linkage*	*II*	
**2**	Pro-Research Environment	18	16	2	*Linkage*	*III*	
**3**	Financial Assistance for Students	14	15	-1	*Linkage*	*III*	
**4**	Effective implementation of rules and regulations	18	15	3	*Linkage*	*IV*	
**5**	Provision of Infrastructure	18	16	2	*Linkage*	*III*	
**6**	Learned and Competent Faculty	17	14	3	*Linkage*	*IV*	
**7**	Availability of State-of-the-Art Technology	18	14	4	*Linkage*	*V*	Key factor but ambivalent
**8**	Industrial Linkages	17	15	2	*Linkage*	*V*	Key factor but ambivalent
**9**	Knowledge Sharing Culture	17	17	0	*Linkage*	*II*	
**10**	Topical Curriculum	10	15	-5	*Linkage*	*II*	
***11***	*Intra-Academia Linkages*	*18*	*15*	*3*	*Linkage*	*VI*	*Key factor*
**12**	Job Placement of Graduates	1	**18**	**-17**	*Dependent*	*I*	
**13**	Faculty Development & Training	18	17	1	*Linkage*	*II*	
**14**	Access to Necessary Business Tools	18	16	2	*Linkage*	*III*	
**15**	Students/Faculty Exchange Program	17	16	1	*Linkage*	*IV*	
**16**	Research Publication Opportunities	17	17	0	*Linkage*	*II*	
**17**	Teacher Student Collaboration	18	17	1	*Linkage*	*II*	
**18**	Business Bodies Accreditation	14	16	-2	*Linkage*	*II*	

The enablers ‘availability of state-of-the-art technology (7)’, ‘industrial linkages (8)’ and ‘intra-academia linkages (11)’ are the critical enablers since they have high driving power and occupy the bottom level (the most critical level) of the model. For the convenience of discerners, these enable have been highlighted grey and italicized in the table.

### 4.2 Discussion

The study’s main objectives are to build an interpretive model by applying classical ISM procedure on the complexity of the relationships of enablers of higher business education, exciting results emerged. Discussion is divided into five parts, i.e., discussion on i) results of the study, ii) contrasting the study with contemporary literature, iii) implications of the study, iv) limitations of the study, and v) recommendations for future studies.

- ***Results of the study*:** Discussion on results is divided into three parts, i.e., discussion on effects of a review of literature, ISM, and MICMAC. The literature review is conducted to prepare a list of enablers for enhancing the quality of business education. As a result, we reached a multitude of enablers that the field experts verified. This list is helpful to draw some structure underlying the phenomenon. ISM is used for modeling. In ISM models, the most critical element(s)/factor(s) (enablers in this study) with high driving power appear at the bottom of the model that is the most critical factor. In the study, ’intra-academia linkages (11)’ ’availability of state-of-the-art technology (7)’ and ’industrial linkages (8)’ occupy the bottom of the model and are the most critical factors. These enablers are the most important and deserve immediate attention from policymakers. The element(s)/factor(s) (enablers in this study) with low driving power appear at the top of the model are the minor critical factor(s). In the study ’job placement of graduates (12)’ occupy top-level of ISM model (*Level I*); enablers appropriate funds for research (1), knowledge sharing culture (9), topical curriculum (10), faculty development & training (13), research publication opportunities (16), teacher-student collaboration (17) and business bodies accreditation (18) occupy upper-middle (*Level II*) these are least critical for policymakers. Other enablers drive these. The element(s)/factor(s) (enablers in this study) with moderate operating power appear in the middle of the model having moderate severity.
- Characteristics at the bottom affect factors in the middle, and factors in the middle affect top-level elements. The severity of effects varies on the continuum of the model’s hierarchy. MICMAC is used for classification and analysis. The objective of MICMAC is to identify critical factors. It classifies the elements of the system into four clusters (autonomous, dependent, linkage, and independent). Characteristics that fall in autonomous clusters have weak driving and weak dependence. These factors are separate from the model, have few but powerful links, and don’t impact the system. The non-existence of elements in autonomous clusters means all factors play an essential role, and the practitioners should pay attention to all aspects. In this study, no enabler is categorized as autonomous. Therefore, all enablers under investigation are relevant and essential. Factors that fall independent have weak driving and strong dependence power; they depend on others and need extra care. Elements having high dependence may also fall in linkage because of high driving at the same time. In the study, ‘job placement of graduates (12)’ is categorized as a dependent driven by others.
- Factors that fall in the linkage cluster have intense driving and strong dependence power. They are unbalanced (action on them affects others, and feedback affects themselves). It means the system is in infancy, and regulators struggle to make sense. In this study, all enablers except ‘job placement of graduates (12)’ fall in linkage cluster that is evidence of an unbalanced/unsettled system. Factors that fall in the independent cluster have high driving power. They may fall in linkage if they have high driving and high dependence at the same time. These are vital factors, and increased care is needed to handle them. Therefore, practitioners should give priority to these enablers. In this study, no such enabler is categorized in this cluster; however, most of the enablers have high driving and, at the same time, high dependence power; therefore, despite their potential of independence, they are categorized as linkage.

The study is comparable to many existing analyses. Results of the study are different from existing literature on various counts, say some variables, method of data collection, identification of relationships, methodology of model building, analysis, and context of the study. The results are therefore compared with highly relevant studies (Table 7).

**Table 7:** Comparison of results of the present study with prior studies.

Study	Focus	Variables	Results	Method
Current	Quality enhancement enablers in higher business education in Pakistan	18 (Table 1)	Key factors are making available state-of-the-art technology and the academic-industrial linkages.	ISM
[62]	Measure the perception and satisfaction level of undergraduate students	3 (Teaching, academic facilities, and physical facilities)	Teaching and educational facilities are key factors.	t-Test and multivariate regression analysis
[63]	Measure the level of service quality in the higher education sector	37	The key factors are administrative, teacher quality, knowledge, leadership quality, and continuous improvement.	Exploratory and confirmatory factor analysis
[44]	Stakeholder collaboration in higher education to improve quality	Four (state agencies, law firms, universities, and students)	Legitimacy is found to be an essential factor.	Case study, open-ended face-to-face, in-depth interview
	Analyze the link between academic enablers and reading achievement measures	Eight (motivation, engagement, standardized test scores, interpersonal skills, reading CBM, study skills, ACES reading skills, and classroom grades)	Classroom grades in particular and others seven are in general critical.	Correlation and simultaneous multiple regression analysis

Our study is comparable with [44, 62, 63]. [60] measured the satisfaction level of undergraduate students regarding teaching, academic facilities, and physical facilities through t-Test and multiple linear regression analysis using primary data of Likert scale. They found that teaching & educational facilities are critical factors [63]. Measured service quality in the higher education sector using exploratory & confirmatory factor analysis. They concluded that administrative services, teacher quality, knowledge services, leadership quality & continuous improvement are key factors [44]. Evaluated stakeholder collaboration in higher education to improve quality through case studies of state agencies, law firms, universities, and students using open-ended face-to-face in-depth interview data and concluded that legitimacy is the critical factor. Jenkins analyzed the link between academic enablers and reading achievement measures using correlation and simultaneous multiple regression and concluded that classroom grades are key factors. The study in hand is different in context, methodology, number of variables covered, type of data used, results, contributions of the research, and practical implications.

- ***Implications of the study*:** The study has substantial practical and theoretical implications:
  ***Practical implications*:** Governmental and/or Regulatory Institutions can benefit from the study’s findings by using it in adjusting the policies regarding the alignment of business education with current requirements of the industry. Since the survey has prioritized quality enhancement enablers, the government or regulators can set their priority actions. They can make informed decisions and better guide academic institutions. The administration of educational institutions and competitors can benefit from the study’s findings by way of adjusting their policies and curriculum accordingly. They can better judge the direction of regulators. Accreditation agencies can benefit from the study results by making informed decisions about the assessment of business schools and their rankings. They can develop better and more informed rubrics and weights for different evaluation criteria. Faculty members can benefit from the study’s findings by giving somewhat knowledgeable inputs to students. Current students, potential students, alumni and/or parents can benefit from the study results by better understanding the priorities and adjusting their preferences accordingly. The general public/local community, media, etc., can benefit from the study’s findings by changing the roles they play towards enhancing the quality of education. The research community can benefit from the study results by developing and adjusting the frameworks of future studies concerning the phenomenon.
  ***Theoretical implications***: The study is the source of enhancing the frontiers of existing literature on enablers of quality enhancement of education in business. It contributes a theoretical model built on a range of enablers about a quality enhancement that also put them into a valuable logical order for the practitioners.
- ***Limitations of the study*:** The study has some data, resources, and methodological limitations like the data in the study are primarily collected in a field setting from a focus group, the generalization, therefore, is accordingly limited. The researchers conduct this research without any financial support in limited available time; consequently, the scope of the study is thus limited, and the methodology used in this study is qualitative, which is appropriate for the study conducted in the context of Pakistan/developing countries; therefore, the generalizability of results is accordingly limited.
- ***Recommendations for future studies*:** We recommend future researchers to i) use quantitative methodologies, e.g., SEM, Wavelet analysis GMM, etc., ii) use Total Interpretive Structural Modeling (Modified, Polarized), etc., iii) use Delphi method or some new methods to create consensus instead of majority rule, iv) constitute the larger size of the panel from other countries, v) use logic-knowledge based questionnaire or other forms of the instrument of measure instead of matrix questionnaire, vi) prepare a more comprehensive list of barriers, vii) design studies using multivalence of data collection, and/or viii) design research studies at the international level duly funded by some intuition.

The study contributed to literature an ISM model of enablers for quality enhancement in higher business education, classification of enablers, and new information about the relationships of enables and discussion on the hierarchy of enablers qua reality.

## 5. Conclusion

By admitting ‘management’ as a science, higher business education has become the fundamental building block of higher education and is ever-important over the period. The research problem under investigation is to identify, analyze, and classify the mystified enablers of higher business education quality enhancement. Therefore, the study’s objectives are to identify, hierarchical, and organize the enablers, build an interpretive model, and discuss the model and its implications, in reality, using literature review, ISM, and MICMAC methods. Results of the literature show that eighteen vital enablers contribute to the phenomenon of quality of higher business education. Results of ISM show that enabler ‘job placement of graduates (12)’ occupy top-level of ISM model (*Level I*); enablers appropriate funds for research (1), knowledge sharing culture (9), topical curriculum (10), faculty development & training (13), research publication opportunities (16), teacher-student collaboration (17) and business bodies accreditation (18) occupy upper-middle (*Level II*); enablers pro-research environment (2), financial assistance for students (3), provision of infrastructure (5) and access to necessary business tools (14) (*Level III*) and enablers effective implementation of rules and regulations (4), learned and competent faculty (6) and students/faculty exchange program (15) occupy middle (*Level IV*); enablers availability of state-of-the-art technology (7) and industrial linkages (8) occupy lower middle (*Level V*), and enabler intra-academia linkages (11) occupy the bottom of the model (*Level VI*). ISM is a bottom-up model (the base is more critical than the top); therefore, factors at the bottom of almost at the bottom need more and immediate attention from policymakers. Results of MICMAC show that all enablers: appropriate funds for research (1), pro-research environment (2), financial assistance for students (3), effective implementation of rules and regulations (4), provision of infrastructure (5), learned and competent faculty (6), availability of state-of-the-art technology (7), industrial linkages (8), knowledge sharing culture (9), topical curriculum (10), intra-academia linkages (11), faculty development & training (13), access to necessary business tools (14), students/faculty exchange program (15), research publication opportunities (16), teacher-student collaboration (17) and business bodies accreditation (18) are classified into linkage cluster, whereas, enabler ‘job placement of graduates (12), is classified into the dependent cluster. No enabler is classified in the independent cluster and autonomous cluster. The study’s overall result shows that the system is unbalanced and unsettled; therefore needs immediate attention from policymakers. The study has valuable theoretical contributions, e.g., identification of enablers for achieving quality in higher business education, ISM model, driving-dependence diagram, a simplified representation of complex relationships among a multitude of enablers of quality, and supplementary statistical/mathematical information in the form of abridged results, comparative discussion, etc. It also has important practical, managerial, and social implications for Higher Education Institutions (HEIs) running business schools, regulators of HEIs, accreditation/ranking bodies for business programs, researchers, students, parents, industry, society at large, and the international community. For HEIs, it helps set priorities to embark on the regime of quality; for regulators, it is helpful in policy-making; for accreditation/ranking bodies, it helps assign weights to each criterion among that of a multitude, for researchers to develop research frameworks for future research, for students, parents, industry, society at the large and international community it helps understand the underlying complex relations of quality of higher business education. Firstly, the ISM method only identifies but does not quantify the relationships; therefore, future studies may use different techniques like SEM, PCA, AHP, ANP, TOPSIS, GRA, etc., to quantify the relations among enablers. Secondly, key enablers have been identified from limited literature; hence might have been overlooked factors; therefore, future studies should explore other relevant factors/variables by using different techniques like PCA or rather thorough literature review and/or analyze the current factors inductive deductive methods. Thirdly, the research advanced some evidence from Pakistan since there are varying cultural, social, technological, and political systems; therefore, generalizing results is limited. Similar future research should be conducted in different contexts and settings. Fourthly, this study is based on data collected from a few critical homogeneous types of stakeholders; therefore, the results’ generalizability is limited. It is highly recommended that future research take inputs from other stakeholders.

## Supporting information

S1 Data (XLSX)

S1 Annex (DOCX)

S2 Annex (DOCX)
