# Supplementary material for: Understanding the interaction among enablers of quality enhancement of higher business education in Pakistan
Source: PLoS One. 2022 May 26;17(5):e0267919. doi: 10.1371/journal.pone.0267919 (PMC9135224; doi:10.1371/journal.pone.0267919)
Supplement: S1 Annex — (DOCX) [file pone.0267919.s002.docx]

***Annexure 1***

**Summarized Questionnaire**

| **Code** | **Enablers** | **1** | **2** | **3** | **4** | **5** | **6** | **7** | **8** | **9** | **10** | **11** | **12** | **13** | **14** | **15** | **16** | **17** | **18** |
| --- | --- | --- | --- | --- | --- | --- | --- | --- | --- | --- | --- | --- | --- | --- | --- | --- | --- | --- | --- |
| **1** | Appropriate Funds for Research |  |  |  |  |  |  |  |  |  |  |  |  |  |  |  |  |  |  |
| **2** | Pro-Research Environment |  |  |  |  |  |  |  |  |  |  |  |  |  |  |  |  |  |  |
| **3** | Financial Assistance for Students |  |  |  |  |  |  |  |  |  |  |  |  |  |  |  |  |  |  |
| **4** | Effective Implementation of rules and regulations |  |  |  |  |  |  |  |  |  |  |  |  |  |  |  |  |  |  |
| **5** | Provision of Infrastructure |  |  |  |  |  |  |  |  |  |  |  |  |  |  |  |  |  |  |
| **6** | Learned and Competent Faculty |  |  |  |  |  |  |  |  |  |  |  |  |  |  |  |  |  |  |
| **7** | Availability of State-of-the-Art Technology |  |  |  |  |  |  |  |  |  |  |  |  |  |  |  |  |  |  |
| **8** | Industrial Linkages |  |  |  |  |  |  |  |  |  |  |  |  |  |  |  |  |  |  |
| **9** | Knowledge Sharing Culture |  |  |  |  |  |  |  |  |  |  |  |  |  |  |  |  |  |  |
| **10** | Topical Curriculum |  |  |  |  |  |  |  |  |  |  |  |  |  |  |  |  |  |  |
| **11** | Intra-Academia Linkages |  |  |  |  |  |  |  |  |  |  |  |  |  |  |  |  |  |  |
| **12** | Job Placement of Graduates |  |  |  |  |  |  |  |  |  |  |  |  |  |  |  |  |  |  |
| **13** | Faculty Development & Training |  |  |  |  |  |  |  |  |  |  |  |  |  |  |  |  |  |  |
| **14** | Access to Necessary Business Tools |  |  |  |  |  |  |  |  |  |  |  |  |  |  |  |  |  |  |
| **15** | Students/Faculty Exchange Program |  |  |  |  |  |  |  |  |  |  |  |  |  |  |  |  |  |  |
| **16** | Research Publication Opportunities |  |  |  |  |  |  |  |  |  |  |  |  |  |  |  |  |  |  |
| **17** | Teacher Student Collaboration |  |  |  |  |  |  |  |  |  |  |  |  |  |  |  |  |  |  |
| **18** | Business Bodies Accreditation |  |  |  |  |  |  |  |  |  |  |  |  |  |  |  |  |  |  |
