# Supplementary material for: Understanding the interaction among enablers of quality enhancement of higher business education in Pakistan
Source: PLoS One. 2022 May 26;17(5):e0267919. doi: 10.1371/journal.pone.0267919 (PMC9135224; doi:10.1371/journal.pone.0267919)
Supplement: S2 Annex — (DOCX) [file pone.0267919.s003.docx]

***Annexure 2***

**Table S1:** Iteration I

| **Code** | **Reachability Set** | **Antecedent Set** | **Intersection Set** | **Level** |
| --- | --- | --- | --- | --- |
| **1** | 1,2,3,4,5,6,7,8,9,10,11,12,13,14,15,16,17,18 | 1,2,3,4,5,6,7,8,9,10,11,13,14,15,16,17,18 | 1,2,3,4,5,6,7,8,9,10,11,13,14,15,16,17,18 |  |
| **2** | 1,2,3,4,5,6,7,8,9,10,11,12,13,14,15,16,17,18 | 1,2,3,4,5,6,7,8,9,11,13,14,15,16,17,18 | 1,2,3,4,5,6,7,8,9,11,13,14,15,16,17,18 |  |
| **3** | 1,2,3,5,7,8,9,11,12,13,14,16,17,18 | 1,2,3,4,5,6,7,8,9,11,13,14,15,16,17 | 1,2,3,5,7,8,9,11,13,14,16,17 |  |
| **4** | 1,2,3,4,5,6,7,8,9,10,11,12,13,14,15,16,17,18 | 1,2,4,5,6,7,8,9,10,11,13,14,15,16,17 | 1,2,4,5,6,7,8,9,10,11,13,14,15,16,17 |  |
| **5** | 1,2,3,4,5,6,7,8,9,10,11,12,13,14,15,16,17,18 | 1,2,3,4,5,6,7,8,9,11,13,14,15,16,17,18 | 1,2,3,4,5,6,7,8,9,11,13,14,15,16,17,18 |  |
| **6** | 1,2,3,4,5,6,8,9,10,11,12,13,14,15,16,17,18 | 1,2,4,5,6,7,8,9,11,13,14,15,16,17 | 1,2,4,5,6,8,9,11,13,14,15,16,17 |  |
| **7** | 1,2,3,4,5,6,7,8,9,10,11,12,13,14,15,16,17,18 | 1,2,3,4,5,7,8,11,13,14,15,16,17,18 | 1,2,3,4,5,7,8,11,13,14,15,16,17,18 |  |
| **8** | 1,2,3,4,5,6,7,8,9,10,12,13,14,15,16,17,18 | 1,2,3,4,5,6,7,8,9,11,13,14,16,17,18 | 1,2,3,4,5,6,7,8,9,13,14,16,17,18 |  |
| **9** | 1,2,3,4,5,6,7,8,10,11,12,13,14,15,16,17,18 | 1,2,3,4,5,6,7,8,9,10,11,13,14,15,16,17,18 | 1,2,3,4,5,6,7,8,10,11,13,14,15,16,17,18 |  |
| **10** | 1,4,9,10,11,12,13,15,16,17 | 1,2,4,5,6,7,8,9,10,11,13,14,15,16,17 | 1,4,9,10,11,13,15,16,17 |  |
| **11** | 1,2,3,4,5,6,7,8,9,10,11,12,13,14,15,16,17,18 | 1,2,3,4,5,6,7,9,10,11,13,14,15,17,18 | 1,2,3,4,5,6,7,9,10,11,13,14,15,17,18 |  |
| **12** | 12 | 1,2,3,4,5,6,7,8,9,10,11,12,13,14,15,16,17,18 | 12 | *I* |
| **13** | 1,2,3,4,5,6,7,8,9,10,11,12,13,14,15,16,17,18 | 1,2,3,4,5,6,7,8,9,10,11,13,14,15,16,17,18 | 1,2,3,4,5,6,7,8,9,10,11,13,14,15,16,17,18 |  |
| **14** | 1,2,3,4,5,6,7,8,9,10,11,12,13,14,15,16,17,18 | 1,2,3,4,5,6,7,8,9,11,13,14,15,16,17,18 | 1,2,3,4,5,6,7,8,9,11,13,14,15,16,17,18 |  |
| **15** | 1,2,3,4,5,6,7,9,10,11,12,13,14,15,16,17,18 | 1,2,4,5,6,7,8,9,10,11,13,14,15,16,17,18 | 1,2,4,5,6,7,9,10,11,13,14,15,16,17,18 |  |
| **16** | 1,2,3,4,5,6,7,8,9,10,12,13,14,15,16,17,18 | 1,2,3,4,5,6,7,8,9,10,11,13,14,15,16,17,18 | 1,2,3,4,5,6,7,8,9,10,13,14,15,16,17,18 |  |
| **17** | 1,2,3,4,5,6,7,8,9,10,11,12,13,14,15,16,17,18 | 1,2,3,4,5,6,7,8,9,10,11,13,14,15,16,17,18 | 1,2,3,4,5,6,7,8,9,10,11,13,14,15,16,17,18 |  |
| **18** | 1,2,5,7,8,9,11,12,13,14,15,16,17,18 | 1,2,3,4,5,6,7,8,9,11,13,14,15,16,17,18 | 1,2,5,7,8,9,11,13,14,15,16,17,18 |  |

**Table S2:** Iteration II

| **Code** | **Reachability Set** | **Antecedent Set** | **Intersection Set** | **Level** |
| --- | --- | --- | --- | --- |
| **1** | 1,2,3,4,5,6,7,8,9,10,11,13,14,15,16,17,18 | 1,2,3,4,5,6,7,8,9,10,11,13,14,15,16,17,18 | 1,2,3,4,5,6,7,8,9,10,11,13,14,15,16,17,18 | *II* |
| **2** | 1,2,3,4,5,6,7,8,9,10,11,13,14,15,16,17,18 | 1,2,3,4,5,6,7,8,9,11,13,14,15,16,17,18 | 1,2,3,4,5,6,7,8,9,11,13,14,15,16,17,18 |  |
| **3** | 1,2,3,5,7,8,9,11,13,14,16,17,18 | 1,2,3,4,5,6,7,8,9,11,13,14,15,16,17 | 1,2,3,5,7,8,9,11,13,14,16,17 |  |
| **4** | 1,2,3,4,5,6,7,8,9,10,11,13,14,15,16,17,18 | 1,2,4,5,6,7,8,9,10,11,13,14,15,16,17 | 1,2,4,5,6,7,8,9,10,11,13,14,15,16,17 |  |
| **5** | 1,2,3,4,5,6,7,8,9,10,11,13,14,15,16,17,18 | 1,2,3,4,5,6,7,8,9,11,13,14,15,16,17,18 | 1,2,3,4,5,6,7,8,9,11,13,14,15,16,17,18 |  |
| **6** | 1,2,3,4,5,6,8,9,10,11,13,14,15,16,17,18 | 1,2,4,5,6,7,8,9,11,13,14,15,16,17 | 1,2,4,5,6,8,9,11,13,14,15,16,17 |  |
| **7** | 1,2,3,4,5,6,7,8,9,10,11,13,14,15,16,17,18 | 1,2,3,4,5,7,8,11,13,14,15,16,17,18 | 1,2,3,4,5,7,8,11,13,14,15,16,17,18 |  |
| **8** | 1,2,3,4,5,6,7,8,9,10,13,14,15,16,17,18 | 1,2,3,4,5,6,7,8,9,11,13,14,16,17,18 | 1,2,3,4,5,6,7,8,9,13,14,16,17,18 |  |
| **9** | 1,2,3,4,5,6,7,8,10,11,13,14,15,16,17,18 | 1,2,3,4,5,6,7,8,9,10,11,13,14,15,16,17,18 | 1,2,3,4,5,6,7,8,10,11,13,14,15,16,17,18 | *II* |
| **10** | 1,4,9,10,11,13,15,16,17 | 1,2,4,5,6,7,8,9,10,11,13,14,15,16,17 | 1,4,9,10,11,13,15,16,17 | *II* |
| **11** | 1,2,3,4,5,6,7,8,9,10,11,13,14,15,16,17,18 | 1,2,3,4,5,6,7,9,10,11,13,14,15,17,18 | 1,2,3,4,5,6,7,9,10,11,13,14,15,17,18 |  |
| **13** | 1,2,3,4,5,6,7,8,9,10,11,13,14,15,16,17,18 | 1,2,3,4,5,6,7,8,9,10,11,13,14,15,16,17,18 | 1,2,3,4,5,6,7,8,9,10,11,13,14,15,16,17,18 | *II* |
| **14** | 1,2,3,4,5,6,7,8,9,10,11,13,14,15,16,17,18 | 1,2,3,4,5,6,7,8,9,11,13,14,15,16,17,18 | 1,2,3,4,5,6,7,8,9,11,13,14,15,16,17,18 |  |
| **15** | 1,2,3,4,5,6,7,9,10,11,13,14,15,16,17,18 | 1,2,4,5,6,7,8,9,10,11,13,14,15,16,17,18 | 1,2,4,5,6,7,9,10,11,13,14,15,16,17,18 |  |
| **16** | 1,2,3,4,5,6,7,8,9,10,13,14,15,16,17,18 | 1,2,3,4,5,6,7,8,9,10,11,13,14,15,16,17,18 | 1,2,3,4,5,6,7,8,9,10,13,14,15,16,17,18 | *II* |
| **17** | 1,2,3,4,5,6,7,8,9,10,11,13,14,15,16,17,18 | 1,2,3,4,5,6,7,8,9,10,11,13,14,15,16,17,18 | 1,2,3,4,5,6,7,8,9,10,11,13,14,15,16,17,18 | *II* |
| **18** | 1,2,5,7,8,9,11,13,14,15,16,17,18 | 1,2,3,4,5,6,7,8,9,11,13,14,15,16,17,18 | 1,2,5,7,8,9,11,13,14,15,16,17,18 | *II* |

**Table S3:** Iteration III

| **Code** | **Reachability Set** | **Antecedent Set** | **Intersection Set** | **Level** |
| --- | --- | --- | --- | --- |
| **2** | 2,3,4,5,6,7,8,11,14,15 | 2,3,4,5,6,7,8,11,14,15 | 2,3,4,5,6,7,8,11,14,15 | *III* |
| **3** | 2,3,5,7,8,11,14 | 2,3,4,5,6,7,8,11,14,15 | 2,3,5,7,8,11,14 | *III* |
| **4** | 2,3,4,5,6,7,8,11,14,15 | 2,4,5,6,7,8,11,14,15 | 2,4,5,6,7,8,11,14,15 |  |
| **5** | 2,3,4,5,6,7,8,11,14,15 | 2,3,4,5,6,7,8,11,14,15 | 2,3,4,5,6,7,8,11,14,15 | *III* |
| **6** | 2,3,4,5,6,8,11,14,15 | 2,4,5,6,7,8,11,14,15 | 2,4,5,6,8,11,14,15 |  |
| **7** | 2,3,4,5,6,7,8,11,14,15 | 2,3,4,5,7,8,11,14,15 | 2,3,4,5,7,8,11,14,15 |  |
| **8** | 2,3,4,5,6,7,8,14,15 | 2,3,4,5,6,7,8,11,14 | 2,3,4,5,6,7,8,14 |  |
| **11** | 2,3,4,5,6,7,8,11,14,15 | 2,3,4,5,6,7,11,14,15 | 2,3,4,5,6,7,11,14,15 |  |
| **14** | 2,3,4,5,6,7,8,11,14,15 | 2,3,4,5,6,7,8,11,14,15 | 2,3,4,5,6,7,8,11,14,15 | *III* |
| **15** | 2,3,4,5,6,7,11,14,15 | 2,4,5,6,7,8,11,14,15 | 2,4,5,6,7,11,14,15 |  |

**Table S4:** Iteration IV

| **Code** | **Reachability Set** | **Antecedent Set** | **Intersection Set** | **Level** |
| --- | --- | --- | --- | --- |
| **4** | 4,6,7,8,11,15 | 4,6,7,8,11,15 | 4,6,7,8,11,15 | *IV* |
| **6** | 4,6,8,11,15 | 4,6,7,8,11,15 | 4,6,8,11,15 | *IV* |
| **7** | 4,6,7,8,11,15 | 4,7,8,11,15 | 4,7,8,11,15 |  |
| **8** | 4,6,7,8,15 | 4,6,7,8,11 | 4,6,7,8 |  |
| **11** | 4,6,7,8,11,15 | 4,6,7,11,15 | 4,6,7,11,15 |  |
| **15** | 4,6,7,11,15 | 4,6,7,8,11,15 | 4,6,7,11,15 | *IV* |

**Table S5:** Iteration V

| **Code** | **Reachability Set** | **Antecedent Set** | **Intersection Set** | **Level** |
| --- | --- | --- | --- | --- |
| **7** | 7,8,11 | 7,8,11 | 7,8,11 | *V* |
| **8** | 7,8 | 7,8,11 | 7,8 | *V* |
| **11** | 7,8,11 | 7,11 | 7,11 |  |

**Table S6:** Iteration VI

| **Code** | **Reachability Set** | **Antecedent Set** | **Intersection Set** | **Level** |
| --- | --- | --- | --- | --- |
| **11** | 11 | 11 | 11 | *VI* |

**Table S7:** Summary of Iterations

| **Code** | **Reachability Set** | **Antecedent Set** | **Intersection Set** | **Level** |
| --- | --- | --- | --- | --- |
| 12 | 12 | 1,2,3,4,5,6,7,8,9,10,11,12,13,14,15,16,17,18 | 12 | I |
| **1** | 1,2,3,4,5,6,7,8,9,10,11,13,14,15,16,17,18 | 1,2,3,4,5,6,7,8,9,10,11,13,14,15,16,17,18 | 1,2,3,4,5,6,7,8,9,10,11,13,14,15,16,17,18 | *II* |
| **9** | 1,2,3,4,5,6,7,8,10,11,13,14,15,16,17,18 | 1,2,3,4,5,6,7,8,9,10,11,13,14,15,16,17,18 | 1,2,3,4,5,6,7,8,10,11,13,14,15,16,17,18 | *II* |
| **10** | 1,4,9,10,11,13,15,16,17 | 1,2,4,5,6,7,8,9,10,11,13,14,15,16,17 | 1,4,9,10,11,13,15,16,17 | *II* |
| **13** | 1,2,3,4,5,6,7,8,9,10,11,13,14,15,16,17,18 | 1,2,3,4,5,6,7,8,9,10,11,13,14,15,16,17,18 | 1,2,3,4,5,6,7,8,9,10,11,13,14,15,16,17,18 | *II* |
| **16** | 1,2,3,4,5,6,7,8,9,10,13,14,15,16,17,18 | 1,2,3,4,5,6,7,8,9,10,11,13,14,15,16,17,18 | 1,2,3,4,5,6,7,8,9,10,13,14,15,16,17,18 | *II* |
| **17** | 1,2,3,4,5,6,7,8,9,10,11,13,14,15,16,17,18 | 1,2,3,4,5,6,7,8,9,10,11,13,14,15,16,17,18 | 1,2,3,4,5,6,7,8,9,10,11,13,14,15,16,17,18 | *II* |
| **18** | 1,2,5,7,8,9,11,13,14,15,16,17,18 | 1,2,3,4,5,6,7,8,9,11,13,14,15,16,17,18 | 1,2,5,7,8,9,11,13,14,15,16,17,18 | *II* |
| **2** | 2,3,4,5,6,7,8,11,14,15 | 2,3,4,5,6,7,8,11,14,15 | 2,3,4,5,6,7,8,11,14,15 | *III* |
| **3** | 2,3,5,7,8,11,14 | 2,3,4,5,6,7,8,11,14,15 | 2,3,5,7,8,11,14 | *III* |
| **5** | 2,3,4,5,6,7,8,11,14,15 | 2,3,4,5,6,7,8,11,14,15 | 2,3,4,5,6,7,8,11,14,15 | *III* |
| **14** | 2,3,4,5,6,7,8,11,14,15 | 2,3,4,5,6,7,8,11,14,15 | 2,3,4,5,6,7,8,11,14,15 | *III* |
| **4** | 4,6,7,8,11,15 | 4,6,7,8,11,15 | 4,6,7,8,11,15 | *IV* |
| **6** | 4,6,8,11,15 | 4,6,7,8,11,15 | 4,6,8,11,15 | *IV* |
| **15** | 4,6,7,11,15 | 4,6,7,8,11,15 | 4,6,7,11,15 | *IV* |
| **7** | 7,8,11 | 7,8,11 | 7,8,11 | *V* |
| **8** | 7,8 | 7,8,11 | 7,8 | *V* |
| **11** | 11 | 11 | 11 | *VI* |

**Table S8:** Conical Matrix

| **Code** | **12** | **1** | **9** | **10** | **13** | **16** | **17** | **18** | **2** | **3** | **5** | **14** | **4** | **6** | **15** | **7** | **8** | **11** |
| --- | --- | --- | --- | --- | --- | --- | --- | --- | --- | --- | --- | --- | --- | --- | --- | --- | --- | --- |
| **12** | 1 | 0 | 0 | 0 | 0 | 0 | 0 | 0 | 0 | 0 | 0 | 0 | 0 | 0 | 0 | 0 | 0 | 0 |
| **1** | 1* | 1 | 1* | 1* | 1 | 1* | 1 | 1* | 1 | 1 | 1 | 1 | 1* | 1* | 1 | 1 | 1* | 1* |
| **9** | 1* | 1* | 1 | 1* | 1* | 1* | 1 | 1* | 1 | 1* | 1* | 1* | 1 | 1 | 1* | 0 | 1* | 1 |
| **10** | 1 | 1* | 1* | 1 | 1* | 1* | 1* | 0 | 0 | 0 | 0 | 0 | 1* | 0 | 1* | 0 | 0 | 1 |
| **13** | 1* | 1* | 1 | 1 | 1 | 1 | 1* | 1* | 1* | 1* | 1* | 1 | 1 | 1 | 1* | 1 | 1 | 1* |
| **16** | 1* | 1 | 1* | 1* | 1 | 1 | 1* | 1 | 1* | 1* | 1* | 1* | 1* | 1* | 1* | 1* | 1* | 0 |
| **17** | 1* | 1 | 1* | 1 | 1* | 1 | 1 | 1* | 1 | 1* | 1* | 1* | 1 | 1 | 1 | 1* | 1* | 1 |
| **18** | 1 | 1* | 1* | 0 | 1* | 1 | 1* | 1 | 1 | 0 | 1 | 1* | 0 | 0 | 1* | 1* | 1* | 1* |
| **2** | 1 | 1* | 1 | 1* | 1 | 1 | 1 | 1 | 1 | 1* | 1* | 1 | 1* | 1* | 1 | 1* | 1* | 1* |
| **3** | 1 | 1* | 1* | 0 | 1* | 1 | 1* | 1 | 1* | 1 | 1* | 1 | 0 | 0 | 0 | 1* | 1* | 1* |
| **5** | 1 | 1* | 1* | 1* | 1 | 1* | 1 | 1* | 1 | 1* | 1 | 1* | 1* | 1* | 1 | 1 | 1 | 1 |
| **14** | 1 | 1* | 1 | 1* | 1* | 1 | 1 | 1 | 1* | 1* | 1* | 1 | 1* | 1* | 1* | 1 | 1 | 1 |
| **4** | 1 | 1* | 1 | 1 | 1* | 1 | 1 | 1* | 1 | 1 | 1* | 1 | 1 | 1 | 1 | 1* | 1* | 1 |
| **6** | 1* | 1 | 1* | 1* | 1* | 1 | 1 | 1* | 1 | 1 | 1 | 1 | 1 | 1 | 1 | 0 | 1 | 1 |
| **15** | 1 | 1 | 1 | 1* | 1* | 1 | 1 | 1* | 1 | 1 | 1* | 1* | 1* | 1* | 1 | 1* | 0 | 1* |
| **7** | 1* | 1* | 1 | 1* | 1* | 1* | 1* | 1 | 1* | 1 | 1* | 1* | 1 | 1* | 1 | 1 | 1 | 1 |
| **8** | 1 | 1* | 1* | 1* | 1 | 1 | 1* | 1 | 1 | 1 | 1* | 1* | 1* | 1* | 1* | 1* | 1 | 0 |
| **11** | 1 | 1 | 1 | 1 | 1 | 1 | 1 | 1* | 1* | 1* | 1* | 1* | 1 | 1* | 1 | 1* | 1* | 1 |
